# Supplementary material for: A MTA2-SATB2 chromatin complex restrains colonic plasticity toward small intestine by retaining HNF4A at colonic chromatin
Source: Nat Commun. 2024 Apr 27;15:3595. doi: 10.1038/s41467-024-47738-y (PMC11055869; doi:10.1038/s41467-024-47738-y)
Supplement: Supplementary file 1 — Supplementary Information [file 41467_2024_47738_MOESM1_ESM.pdf]

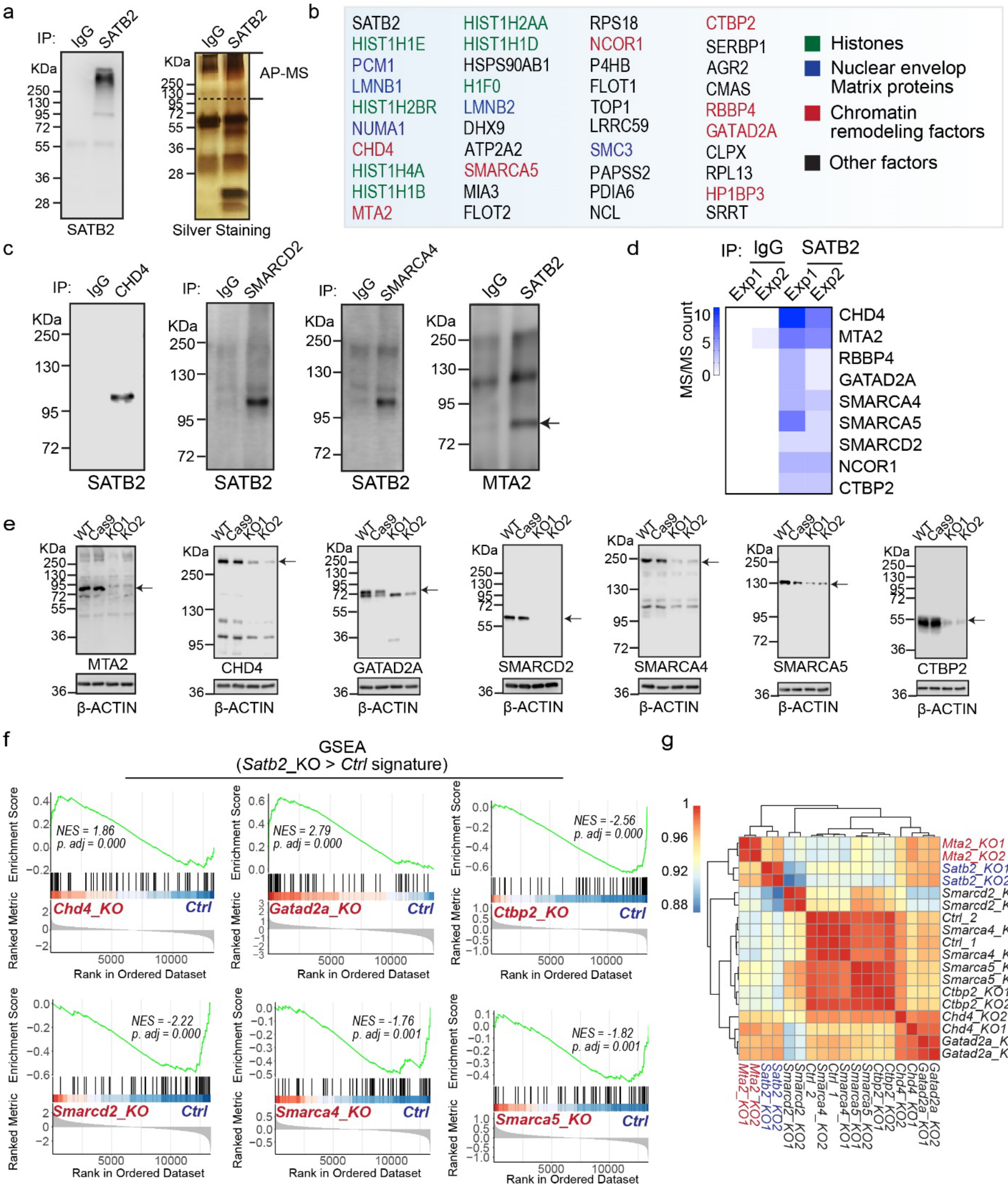

Supplementary Fig. 1. See next page for caption.

Supplementary Fig. 1. AP-MS and CRISPR screen identified candidate SATB2-associated factors that regulate colonic transcription.

(a) Immunoblot of cross-linked murine colonic tissues probed with anti-SATB2 antibody revealed protein complexes at high molecular weight (left panel). Right panel shows silver stain of protein gel loaded with control IgG or anti-SATB2 immunoprecipitated colonic protein samples. The high molecular weight portion of the gel (above the dotted line) was excised for Mass Spectrometry.

(b) The top 40 enriched proteins from two independent AP-MS experiments.

(c) Co-IP with murine colonic tissues showed physical interactions between SATB2 and several of the chromatin remodeling factors identified in AP-MS.

(d) A heatmap of MS counts of the nine chromatin remodeling factors that we selected for CRISPR study. Source data are provided as a Source Data file.

(e) We used CRISPR-CAS9 to disrupt seven chromatin remodeling factors in cultured murine colonic organoids. Immunoblot of two independent experiments, together with two different controls (unmanipulated colonic organoids or colonic organoids transduced with CAS9 but no gRNA), showed successful disruption of each factor. All the different gels/blots were derived from the same experiment and were processed in parallel.

(f) GSEA analysis of RNA-seq data from colonic organoids in which different chromatin remodeling factors were knocked out. Genes up-regulated in Satb2 knockout vs control colonic organoids were used as the gene set for comparison. Positive correlation was seen for Chd4 and Gatad2a, but not Smarcd2, Smarca4, Smarca5, or ctbp2 knockout organoids.

(g) Pearson correlation plot of transcriptomes from different CRISPR knockout organoids and controls. Mta2 knockout transcriptome had a stronger correlation with Satb2 knockout transcriptomes than the other samples.

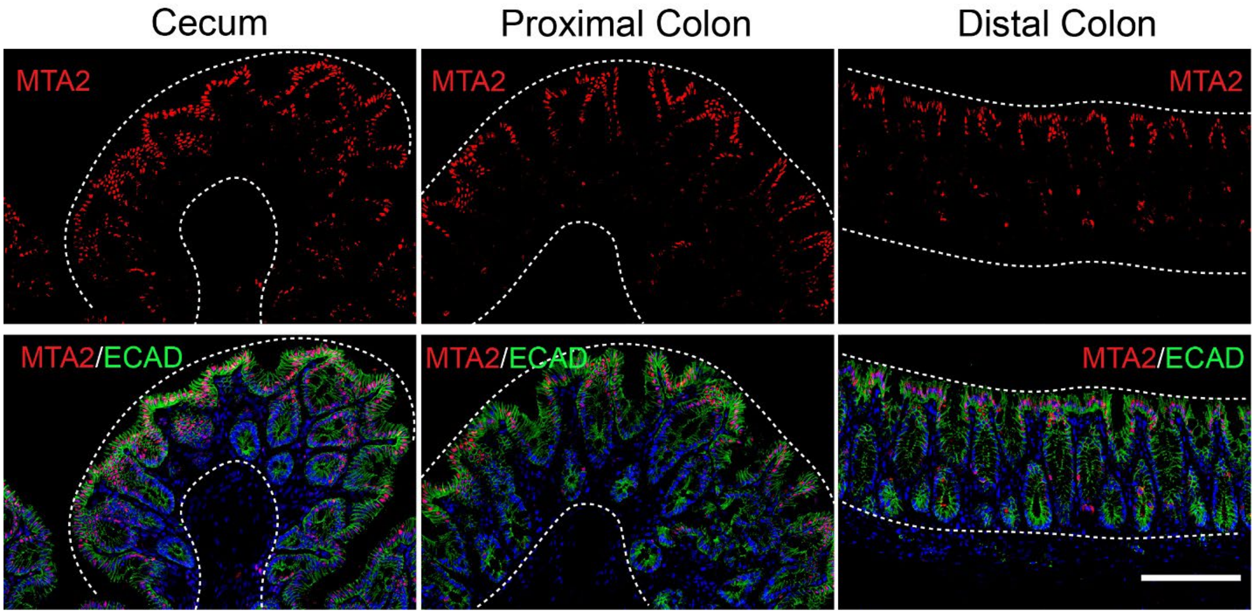

Supplementary Fig. 2. MTA2 is expressed in the upper glands of adult mouse colon.

Immunofluorescent staining of MTA2 in different regions of the adult mouse large intestine showed a predominant localization in the upper glands. Some non-epithelial cells also expressed MTA2. Scale bar = 100 μm. Two independent experiments was repeated with similar results.

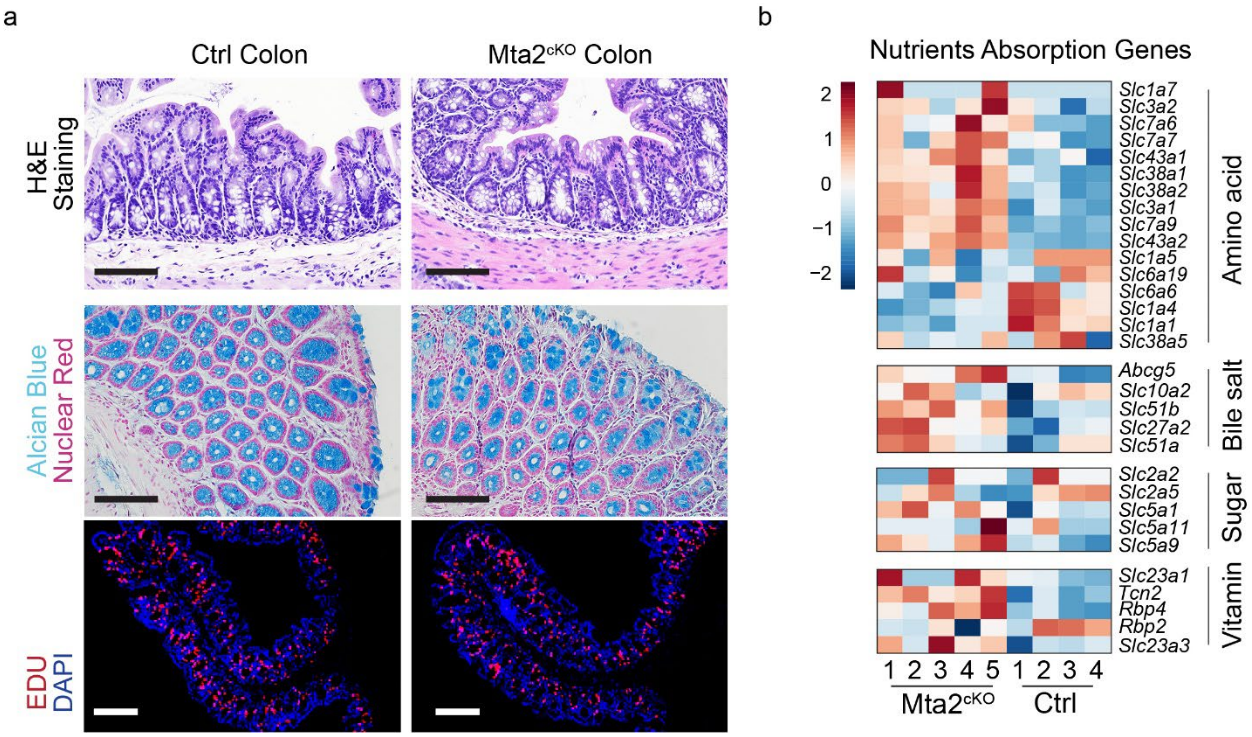

Supplementary Fig. 3. Analysis of Mta2^{cKO} colon.

(a) Mta2^{cKO} colon appeared normal compared with controls, based on H & E stain, Alcian blue stain for goblet cells, and EdU labeling of the proliferative zone. Scale bar = 100 μm. Two independent experiments was repeated with similar results.

(b) Heat map of normalized RNA-seq counts of nutrient transporter genes in control and Mta2^{cKO} colon.

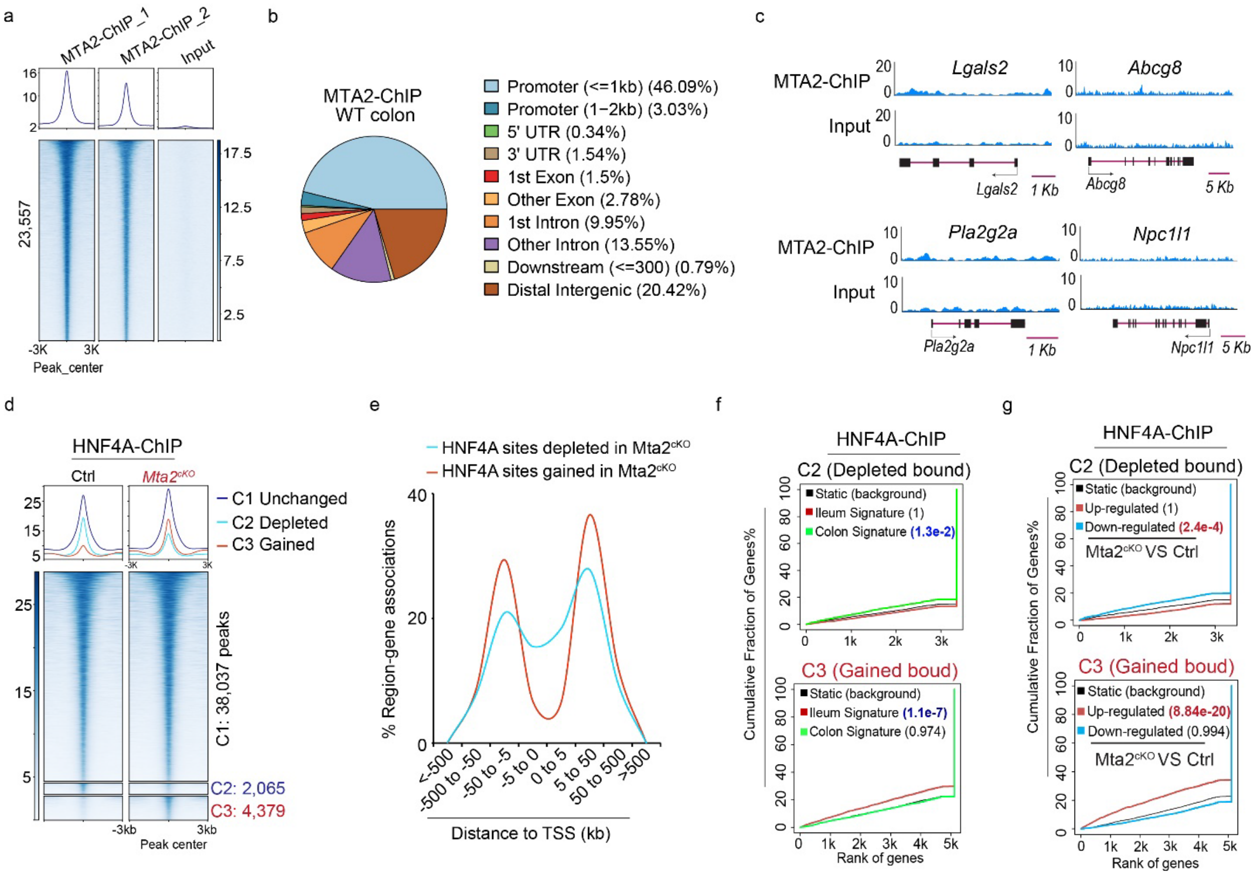

Supplementary Fig. 4. See next page for caption.

Supplementary Fig. 4. MTA2 regulates HNF4A binding in colon

(a, b) MTA2 genomic binding sites in colonic tissues were identified by ChIP-seq using input DNA as controls, yielding 23,557 peaks (peak call by MACS2, duplicate biological samples) (a). The binding sites include distal elements as well as promoters (b).

(c) Genome Browser tracks showing a lack of MTA2 binding at genomic loci of 4 small intestine genes.

(d) DNA binding profiles of HNF4A sites that were unchanged (C1, 38,037 sites), reduced (C2, 2,065 sites), or gained (C3, 4,379 sites) after MTA2 loss in the colon. Plots are 6-kb windows centered on each HNF4A binding site.

(e) DNA binding density plots of HNF4A sites that were depleted (2,065 sites) or gained (4,379 sites) after MTA2 loss in colon. TSS: translation start site.

(f, g) Predictions of enhancer regulatory functions by BETA (binding and expression target analysis) indicate that loss of HNF4A binding in $Mta2^{cKO}$ colon is associated with down-regulation of colonic genes (f, g, upper plots), whereas gain of HNF4A binding is associated with up-regulation of ileal genes (f, g, lower plots). Plots depict the cumulative score of regulatory potential for every gene based on enhancer distances from the TSS. Blue lines represent the background of unaltered genes, and p values denote the significance of up or down associations relative to the background.

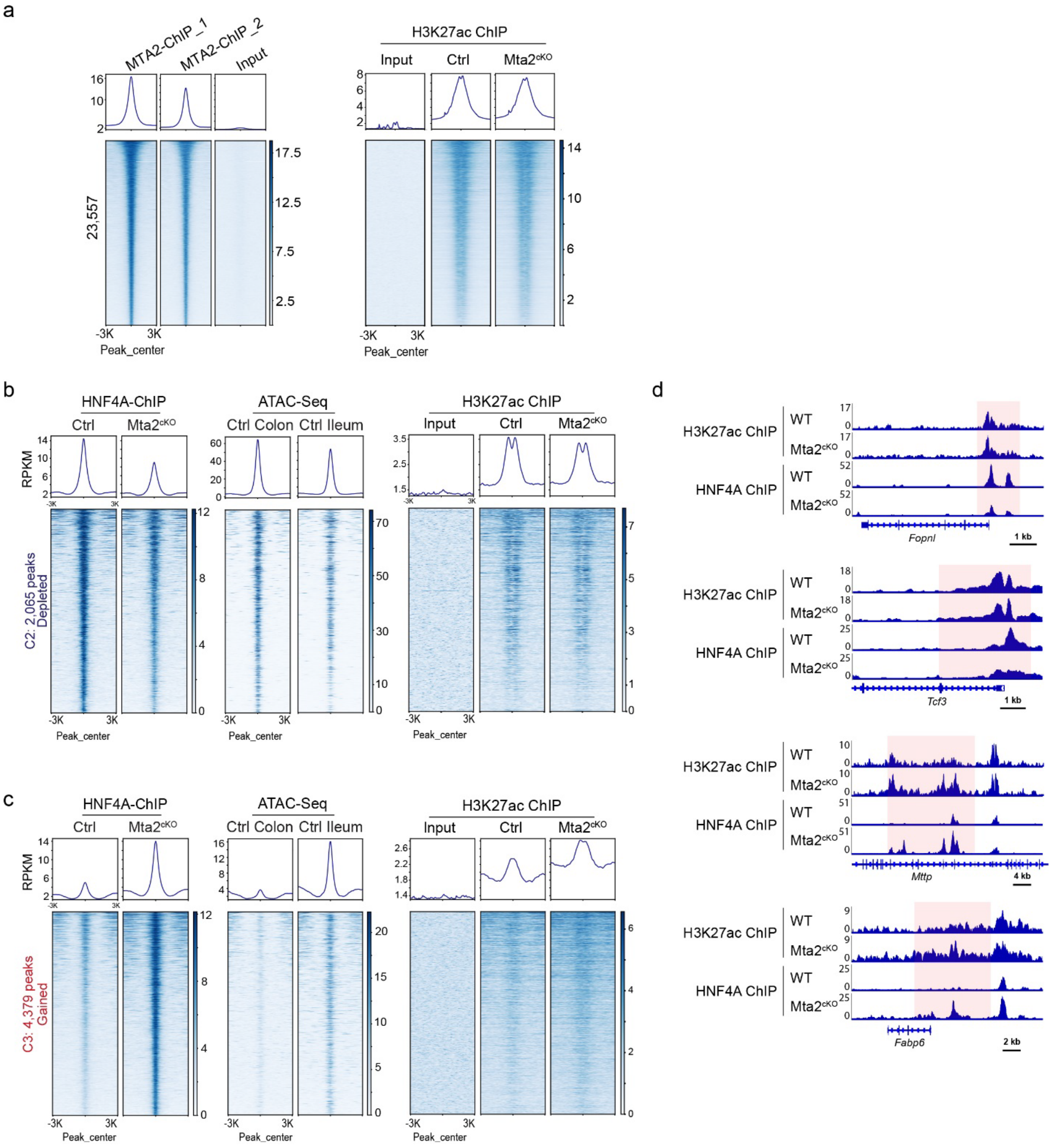

Supplementary Fig. 5. See next page for caption.

Supplementary Fig. 5. Ectopic HNF4A expression in mouse colonic organoids activated small intestine genes

(a) MTA2 genomic binding profiles in colonic tissues (left panel, same as Supplementary Fig. 4a) and the corresponding H3K27ac profiles (right panel).

(b, c) DNA binding profiles of HNF4A sites that were either reduced (b) or gained (c) after MTA2 loss in the colon. Corresponding ATAC signals (in the colon or ileum) and H3K27ac signals were shown. Plots are 6-kb windows centered on each HNF4A binding site. The HNF4A-ChIP and ATAC-seq data shown here are the same as the ones in Fig. 4d.

(d) Genomic browser tracks showing no difference in H3K27ac at two genes with depleted HNF4A binding in $Mta2^{cKO}$ colon, but increased H3K27ac at two genes with enhanced HNF4A binding.

Supplementary Figure 1C Uncropped Image

Red lined area are presented in figure S1C

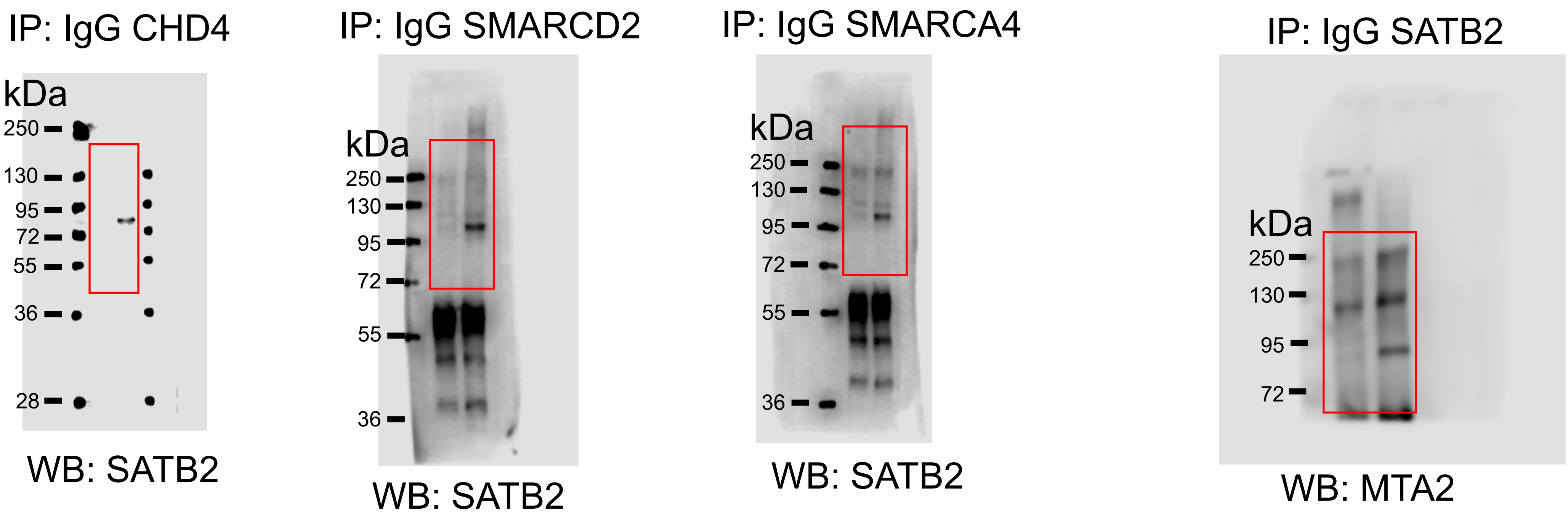

Supplementary Figure 1E Uncropped Image

Red lined area are presented in figure S1E

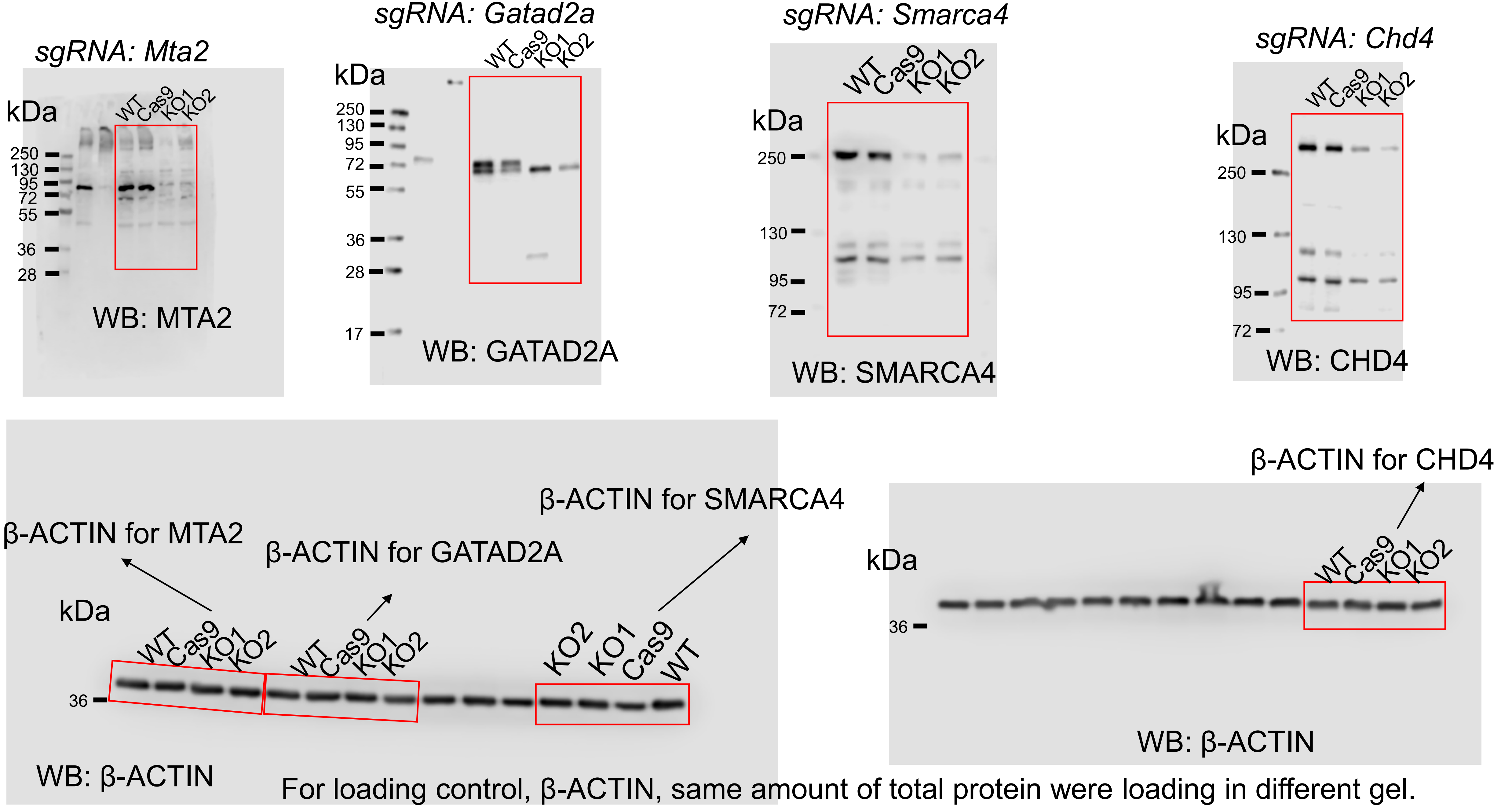

For loading control, β-ACTIN, same amount of total protein were loading in different gel.

Supplementary Figure 1E Uncropped Image

Red lined area are presented in figure S1E

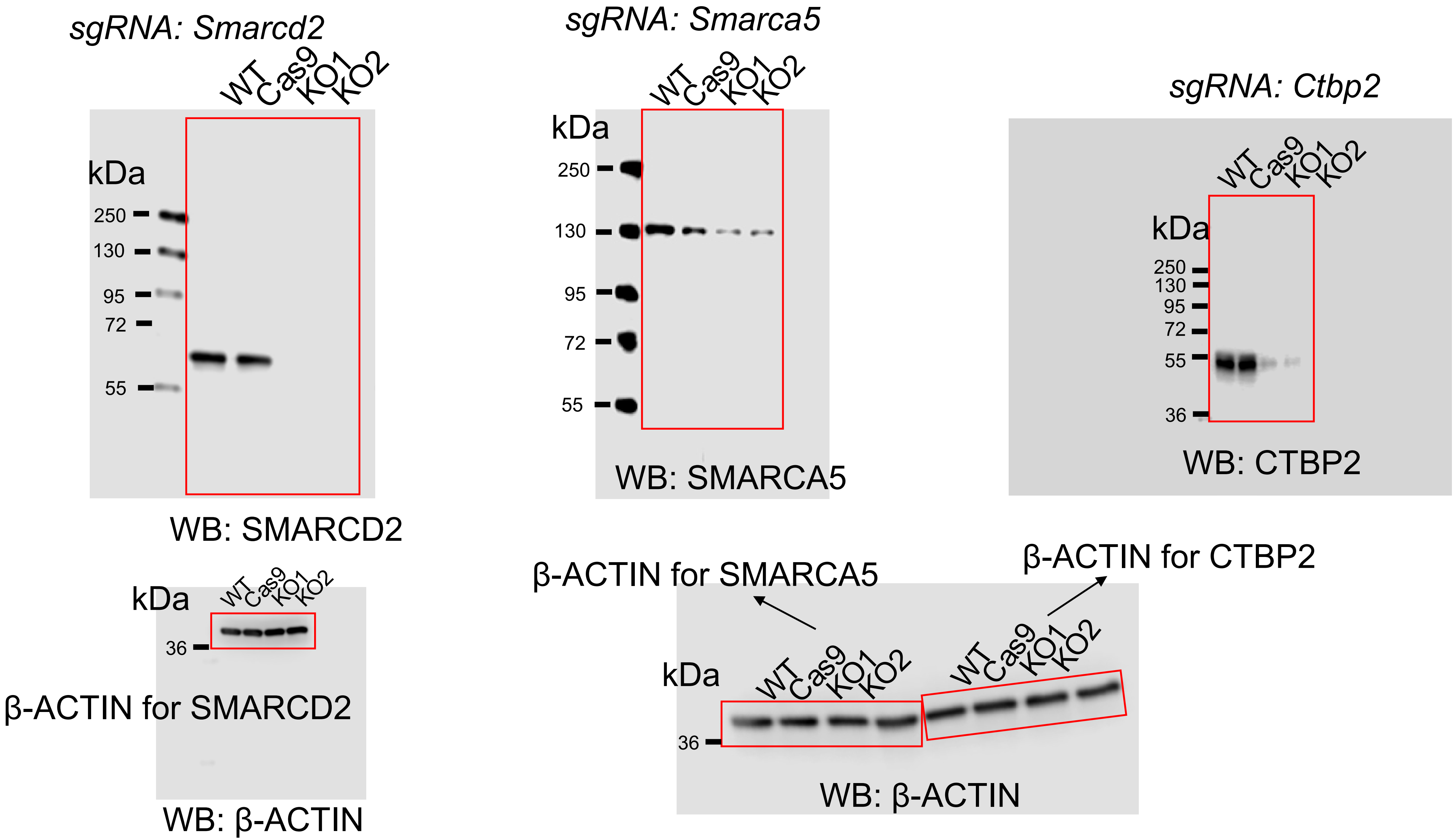

For loading control, β-ACTIN, same amount of total protein were loading in different gel.

Supplementary Table 1. Mouse and Human Colonoid Culture Medium

Mouse Large Intestine Culture Medium (Wnta3-EGF-Noggin-Rspondin1 medium(WENR))

Reagents	Final Conc.
Glutamax	2 mM
HEPES	10 mM
Primocin	100 µg/ml
EGF	50 ng/ml
N2 supplement	1X
B27 supplement	1X
N-acetyl-L-cysteine	1 mM
L -WRN Condition Medium &	50%V/V
A83-01 *	500 nM
Y-27632 *	10 µM
In Advanced DMEM/F12	

& L -WRN cells was originally made in Thaddeus Stappenbeck Lab which secrete Wnt3a, R spondin and Noggin, collected in culture with 20% FBS in Advanced DMEM/F12 for 4 days, filtered and stored in -80°C.

* To avoid anoikis, supplement the culture medium with Y-27632 for the first two days. Supplement A83-01 in WENR for first two passaging.

Human 3D Organoid Culture Medium (HCM)

Reagents	Final Conc.
Glutamax	2 mM
HEPES	10 mM
Primocin	100 µg/ml
EGF	50 ng/ml
N2 supplement	1X
B27 supplement	1X
N-acetyl-L-cysteine	1 mM
L -WRN Condition Medium &	50%V/V
SB202190	10 µM
A83-01	500 nM
Y-27632 *	10 µM
Gastrin	10 nM
Nicotinamide	10 mM
CHIR99021	2.5 µM
In Advanced DMEM/F12	

Human 3D Organoid Differentiation Medium (HDM)

Reagents	Final Conc.
Glutamax	2 mM
HEPES	10 mM
Primocin	100 µg/ml
Y-27632	10 µM
A83-01	500 nM
B27 supplement	1X
N-acetyl-L-cysteine	1 mM
L -WRN Condition Medium &	25%V/V
Gastrin	10 nM
FGF-2	50 ng/ml
IGF-1	100 ng/ml
DAPT #	5 µM
In Advanced DMEM/F12	

DAPT was added for secretion cells niche differentiation (Goblet cells et., al.)

Supplementary Table 2. SATB2 Domain Deletion Oligos and sgRNA Information

Primer_name	Sequence (5'-3')
SATB2_ULT-del_F	TGTTCAAAGTTGGAAGACTT
SATB2_ULT-del_R	TCCCACGGCCTTGGCCACGG
SATB2_CUT1L-del-R	CAAGTCTTCCAACTTTGAAC
SATB2_CUT1L-del-F	AAAGTGGAAAGAGTGGAGCG
SATB2_CUT1-del-R	TGTTGGCTCTGGCTTAACTG
SATB2_CUT1-del-F	CGTGATCGCATTTACCAGGA
SATB2_CUT2-del-R	GTTGGCGCCGTCCACCTTAA
SATB2_CUT2-del-F	CGGGATGTGATCTATGAGGA
SATB2_HOX-del-R	TTTGGCACAGCTGTCTTCTG
SATB2_HOX-del-F	CACCATGGCAAGCTGAAGGA
Target	sgRNA_sequence (5'-3')
Gatad2a	GAGCACAATCACATCAGGCG
Chd4	CCCCTGCTCAGCGGGCAGTG
Mta2	TTACCTGGTTAGACGGATTG
Smarca5	GTATTTGATCATGGATCACC
Smarca4	CAACATGCATCAGATGCACA
Smarcd2	CGGAGCAGCTGTGCCAAATG
Ctbp2	AGGGGGCCGTTCATGATCTG
HDAC1	AAAGTCTGTTACTACTACGA
HDAC2	TGAGTCATCCGGATTCTATG

Supplementary Table 3. Recipe of RIPA (0.3M)

Reagent	Final Concentration
Sodium Dodecyl Sulfate (SDS)	0.1% (w/v)
TritonX-100	1% (w/v)
Sodium Deoxycholate	0.1% (w/v)
Tris-HCl (PH 7.4)	10 mM
EDTA (PH 8.0)	1 mM
Sodium Chloride (NaCl)	0.3 M
MilliQ Water	Adjust
