# Supplementary material for: A MTA2-SATB2 chromatin complex restrains colonic plasticity toward small intestine by retaining HNF4A at colonic chromatin
Source: Nat Commun. 2024 Apr 27;15:3595. doi: 10.1038/s41467-024-47738-y (PMC11055869; doi:10.1038/s41467-024-47738-y)
Supplement: Supplementary file 3 — Description of Additional Supplementary Files [file 41467_2024_47738_MOESM3_ESM.pdf]

Description of Additional Supplementary Files

File Name: Supplementary Data 1
Description: The Data of AP-MS and the SATB2-interacting Protein List

File Name: Supplementary Data 2
Description: Satb2 Knockout Signature Gene List

File Name: Supplementary Data 3
Description: Differential Expression Analysis Table (Mta2cKO vs Control)

File Name: Supplementary Data 4
Description: Genes Proximal to MTA2 Binding Sites in Colon (< 50 kb)

File Name: Supplementary Data 5
Description: SATB2-Interacting Proteins in Colon (MTA2 null vs control) Identified by Mass Spectrometry

File Name: Supplementary Data 6
Description: Reagent Information
