# Supplementary material for: A MTA2-SATB2 chromatin complex restrains colonic plasticity toward small intestine by retaining HNF4A at colonic chromatin
Source: Nat Commun. 2024 Apr 27;15:3595. doi: 10.1038/s41467-024-47738-y (PMC11055869; doi:10.1038/s41467-024-47738-y)
Supplement: Supplementary file 10 — Reporting Summary [file 41467_2024_47738_MOESM10_ESM.pdf]

nature portfolio

Corresponding author(s): Wei Gu and Qiao Zhou

Last updated by author(s): Mar 7, 2024

Reporting Summary

Nature Portfolio wishes to improve the reproducibility of the work that we publish. This form provides structure for consistency and transparency in reporting. For further information on Nature Portfolio policies, see our Editorial Policies and the Editorial Policy Checklist.

Statistics

For all statistical analyses, confirm that the following items are present in the figure legend, table legend, main text, or Methods section.

n/a	Confirmed	
☐	☒	The exact sample size (n) for each experimental group/condition, given as a discrete number and unit of measurement
☐	☒	A statement on whether measurements were taken from distinct samples or whether the same sample was measured repeatedly
☐	☒	The statistical test(s) used AND whether they are one- or two-sided<br>*Only common tests should be described solely by name; describe more complex techniques in the Methods section.*
☐	☒	A description of all covariates tested
☐	☒	A description of any assumptions or corrections, such as tests of normality and adjustment for multiple comparisons
☐	☒	A full description of the statistical parameters including central tendency (e.g. means) or other basic estimates (e.g. regression coefficient) AND variation (e.g. standard deviation) or associated estimates of uncertainty (e.g. confidence intervals)
☐	☒	For null hypothesis testing, the test statistic (e.g. F, t, r) with confidence intervals, effect sizes, degrees of freedom and P value noted<br>*Give P values as exact values whenever suitable.*
☒	☐	For Bayesian analysis, information on the choice of priors and Markov chain Monte Carlo settings
☒	☐	For hierarchical and complex designs, identification of the appropriate level for tests and full reporting of outcomes
☐	☒	Estimates of effect sizes (e.g. Cohen's d, Pearson's r), indicating how they were calculated

Our web collection on statistics for biologists contains articles on many of the points above.

Software and code

Policy information about availability of computer code

Data collection	Prism 9 (9.3.1), R (4.3).

Data analysis	Statistical analysis were done using GraphPad Prism 9 or R 4.3.<br>Bulk RNA-seq analsysis was performed as follow. Briefly, reads alignment to the mouse (mm10) or human (hg38) reference genome was performed by STAR (2.7.5b) package. The raw count tables were generated by featureCounts (2.0.1). The DEseq2 package was used for differential expression analysis. In DESeq2 (1.28.1), the p-values attained by the Wald test are corrected for multiple testing using the Benjamini and Hochberg (BH) method. The Limma (3.44.3) package was used to remove donor-donor variance and batch-effect. Differentially expressed genes were generally determined using parameters of adjusted p-value < 0.05 and LFC > 1 or < -1 unless specified in figure legends. The heatmaps were plotted using the R package, pheatmap (1.0.12). GSEA KEGG analysis and GSEA analysis were conducted with the clusterProfiler package (4.8.1).<br>For ChIP-seq analysis, all reads were trimmed by Trim Galore! (0.6.5) (https://www.bioinformatics.babraham.ac.uk/projects/trim_galore/), and subject to quality control with FastQC (0.12.0) before and after adapter trimming. Briefly, Reads were mapped using Bowtie246 (2.4.1) to mm10 genome; Peaks were called using callpeak function in the MACS247 package (2.2.7.1) with the following parameters (callpeak -t ChIPfile.bam -c Inputfile.bam --format Paired-end BAM --gsize M.musculs(1.87e9) -m 10 30 -q 0.001-bw 300); Bam files were converted into signal files (bigWig) using deepTools v3.4.346,48 . Signals (bigWig) across samples were quantile normalized with haystack v 0.5.549 using 50-bp window across the genome to visualize read distribution on Integrated Genomics Viewer v 2.16.250; BETA -p ChIP_file.bed -e gene_exp.diff -g mm10 -d 50000. We used peaks showing q-val < 0.001 and mfold enrichment 10 to 30 and used ChIP-seq from Inputs or knockouts as controls. BETA (1.0.7Wang et al. 2013) was used to associate genes with HNF4A-ChIP depleted or gained bound sites and quantify these associations using peaks within 50-kb from TSS, a significance threshold of FDR-adjusted P < 0.01 for differential gene expression in wild-type ileum vs. colon or Mta2cKO vs. Ctrl colon, and other default parameters.

For manuscripts utilizing custom algorithms or software that are central to the research but not yet described in published literature, software must be made available to editors and reviewers. We strongly encourage code deposition in a community repository (e.g. GitHub). See the Nature Portfolio guidelines for submitting code & software for further information.

Data

Policy information about availability of data

All manuscripts must include a data availability statement. This statement should provide the following information, where applicable:

- Accession codes, unique identifiers, or web links for publicly available datasets
- A description of any restrictions on data availability
- For clinical datasets or third party data, please ensure that the statement adheres to our policy

The high -throughput sequencing raw and processed data have been deposited to Gene Expression Omnibus (GEO). Bulk RNA-Seq: GSE213879, GSE213878 and GSE245288. ChIP-Seq: GSE213877 and GSE245751. We also analyzed our previously published GEO datasets: GSE148690, GSE167283, GSE167287, and GSE167284. Human (hg38) or mouse (mm10) reference genome sequences used in our study can be found at Gencode GRCh38_v29 or GRCm38_vM20 respectively. All data are available in the main text or the supplementary materials. Source data are provided with this paper.

Research involving human participants, their data, or biological material

Policy information about studies with human participants or human data. See also policy information about sex, gender (identity/presentation), and sexual orientation and race, ethnicity and racism.

Reporting on sex and gender	For HNF4A over-expression in human colon organoid, following human samples were used in the studies:<br>Prep80 (Normal Ascending Colon Organoid) University of Michigan 29Y, Female<br>Prep83 (Normal Ascending Colon Organoid) University of Michigan 45Y, Female<br>Prep87 (Normal Ascending Colon Organoid) University of Michigan 21Y, Male<br>Prep88 (Normal Ascending Colon Organoid) University of Michigan 33Y, Female<br>Prep89 (Normal Ascending Colon Organoid) University of Michigan 55Y, Male
Reporting on race, ethnicity, or other socially relevant groupings	n/a
Population characteristics	n/a
Recruitment	n/a
Ethics oversight	Weill Cornell Medicine

Note that full information on the approval of the study protocol must also be provided in the manuscript.

Field-specific reporting

Please select the one below that is the best fit for your research. If you are not sure, read the appropriate sections before making your selection.

☒ Life sciences ☐ Behavioural & social sciences ☐ Ecological, evolutionary & environmental sciences

For a reference copy of the document with all sections, see nature.com/documents/nr-reporting-summary-flat.pdf

Life sciences study design

All studies must disclose on these points even when the disclosure is negative.

Sample size	No statistical method was performed to predetermine the sample size. We selected the sample size based on rationale of feasibility of experiment, availability of samples and according to the published literature in the same field. Sample size for each experiment was stated in the figure legends.
Data exclusions	No data were excluded
Replication	Most of the experiments were successfully repeated at least 2 times with one exception. (In this study, we performed only one time SATB2-ChIP and HNF4A-ChIP analysis (GSE213877) in colon epithelium cells from both MTA2KO and Wild type mouse. However, we validated the similar called peaks by SATB2 or HNF4A ChIP-seq in wild type mouse from our previously published paper (Gu, W. et al, 2021.) and this study. All other transcript factors and histone-ChIP experiments were successfully repeated 2 times.)<br>All Mass Spectrometry experiments were successfully repeated in 2 different biological samples.<br>All the staining were performed on a minimum of 2 different biological samples and all attempts at replication were successful. For the MTA2 expression in different epithelium lineage, to gain a clear conclusion, we performed experiments on 7 different colon fragments from 3 independent mice as shown in Fig2. Exact repeat number was stated in the figure legends for each experiment.
Randomization	Male and female mice with Mta2cKO (Vil-CreERT2; Mta2f/f) or Ctrl (Mta2f/f) genotype were randomly injected with tamoxifen. Experimental randomization only was applied when pick up mice from same genotype, but not applied to the purpose for the experiments comparing between genotype, as the genotype was assigned by born.
Blinding	The mouse experiments were performed not blinded as the mice assigned to the treatment groups by genotype results. For the in vitro organoid experiment, blinding was not possible , as the assigned treatment such as CRIPSR Knock out was known to the researchers.

Reporting for specific materials, systems and methods

We require information from authors about some types of materials, experimental systems and methods used in many studies. Here, indicate whether each material, system or method listed is relevant to your study. If you are not sure if a list item applies to your research, read the appropriate section before selecting a response.

Materials & experimental systems

n/a	Involved in the study	
☐	☒	Antibodies
☐	☒	Eukaryotic cell lines
☒	☐	Palaeontology and archaeology
☐	☒	Animals and other organisms
☒	☐	Clinical data
☒	☐	Dual use research of concern
☒	☐	Plants

Methods

n/a	Involved in the study	
☐	☒	ChIP-seq
☒	☐	Flow cytometry
☒	☐	MRI-based neuroimaging

Antibodies

Antibodies used

Rabbit polyclonal anti FABP6, Abcam, Cat# ab91184, used for IF (1:500) (RRID: AB_10563324)
Rabbit polyclonal anti CTBP2, Cell Signaling Technology, Cat# 13256, used for WB (1:1000) (RRID: AB_2798164)
Rabbit polyclonal anti SMARCA4, Bethyl Laboratories, Cat# A300-813A, used for WB (1:1000) (RRID: AB_2191850)
Rabbit polyclonal anti MTA2, Abcam, Cat# ab8106, used for WB (1:1000), IF (1:500), IP (1:200), CHIP (1:200) and IHC (1:1000, PH6.0 antigen retrieval) (RRID: AB_306276)
Rabbit polyclonal anti CHD4, Abcam Cat# ab72418, used for WB (1:1000) and IP (1:100) (RRID: AB_1268107)
Rabbit monoclonal anti CHD4, Cell Signaling Technology, Cat# 12011, used for IF (1:500) (RRID: AB_2734702)
Rabbit polyclonal anti P66alpha (GATAD2A), Bethyl Laboratories, Cat# A302-358A, used for WB (1:1000) (RRID: AB_1907236)
Rabbit polyclonal anti SNF2h/ISWI (SMARCA5), Bethyl Laboratories, Cat# A301-017A, used for WB (1:1000) (RRID: AB_2239528)
Rabbit monoclonal anti SMARCD2, Abcam, Cat# ab220164, used for WB (1:1000) (RRID: AB_2904257)
Rabbit polyclonal anti HDAC1, Bethyl Laboratories, Cat# A300-713A, used for WB (1:1000) (RRID: AB_533395)
Rabbit monoclonal anti HDAC1, Cell Signaling Technology, Cat# 34589, used for IP (1:100) (RRID: AB_2756821)
Rabbit polyclonal anti HDAC2, Bethyl Laboratories, Cat# A300-705A, used for WB (1:1000) (RRID: AB_533399)
Rabbit polyclonal anti HDAC2, Cell Signaling Technology, Cat# 2545, used for IP (1:100) (RRID: AB_2116819)
Rabbit monoclonal anti SATB2, Abcam, Cat# ab92446, used for WB (1:2000), CHIP (1:200), IF (1:2000) and IP (1:200) (RRID: AB_10563678)
Mouse monoclonal anti SATB2, Santa Cruz Biotechnology, Cat# sc-518006, used for WB (1:400) (RRID: AB_2904537)
Rabbit monoclonal anti CA1, Abcam, Cat# Ab108367, used for IF (1:1000) (RRID: AB_10863424)
Mouse monoclonal anti FLAG Agarose Gel, Sigma-Aldrich, Cat# A2220, used for IP (30µL Agarose for 500µL lysate) RRID: AB_10063035
Mouse monoclonal anti V5 Tag, Invitrogen, Cat# R960-25, used for WB (1:1000) (RRID: AB_2556564)
Rabbit monoclonal anti HA Tag, Cell Signaling Technology, Cat# 3724, used for WB (1:1000) (RRID: AB_1549585)
Rabbit polyclonal anti MTTP, Sigma-Aldrich, Cat# HPA054862, used for IHC (1:1000) (RRID: AB_2682628)

Mouse monoclonal anti HNF4A, Abcam, Cat# ab41898, used for CHIP (1:200) (RRID: AB_732976)
RAT IgG2a monoclonal anti E-cadherin, Thermo Fisher Scientific, Cat# 13-1900, used for IF (1:3000) (RRID: AB_2533005)
Goat anti Rabbit IgG-HRP (WB), Cell Signaling Technology, Cat# 7074, used for WB (1:3000) (RRID: AB_2099233)
Horse anti Mouse IgG-HRP (WB), Cell Signaling Technology, Cat# 7076, used for WB (1:3000) (RRID: AB_330924)
Goat anti-Rabbit IgG ImmPRESS-HRP Polymer, Vector Laboratories, Cat# MP-7451, used for IHC (No dilution, ready for use) (RRID: AB_2631198)
Rabbit polyclonal anti H3K27Ac, Active Motif, Cat# 39135, used for CHIP (1:200) (RRID: AB_2614979)

Validation

Each antibody has been validated by the companies and by the result of papers.
Anti-FABP6 (Abcam, Cat# ab91184) was used for IF, and suitable for mice, which had been validated at Gu, W. et al, 2021.
Anti-CTBP2(Cell Signaling Technology, Cat# 13256) was used for WB, and suitable for mice, which had been validated by Cell Signaling Technology.
Anti-SMARCA4 (Bethyl Laboratories, Cat# A300-813A) was used for WB, and suitable for mice, which had been validated by Bethyl Laboratories.
Anti-MTA2 (Abcam, Cat# ab8106) was used for WB, IF, IP, IHC, and suitable for mice, which had been validated by Abcam. Application of CHIP was validated in our studies.
Anti-CHD4 (Abcam, Cat# ab72418), used for WB and IP, was validated in human by company. The application in mice was validated in our studies.
Anti-CHD4 (Cell Signaling Technology, Cat# 12011) was used for IF, and suitable for mice, which had been validated by company.
Anti-P66alpha (GATAD2A) (Bethyl Laboratories, Cat# A302-358A) was used for WB, and suitable for mice, which had been validated by Bethyl Laboratories.
Anti-SNF2h/ISWI (SMARCA5) (Bethyl Laboratories, Cat# A301-017A), used for WB, was validated in human by company. The application in mice was validated in our studies.
Anti-SMARCD2 (Abcam, Cat# ab220164) was used for WB, and suitable for mice, which had been validated by Abcam.
Anti-HDAC1 (Bethyl Laboratories, Cat# A300-713A) was used for WB, and suitable for mice, which had been validated by Bethyl Laboratories.
Anti-HDAC1 (Cell Signaling Technology, Cat# 34589) was used for IP, and suitable for mice, which had been validated by company.
Anti-HDAC2 (Bethyl Laboratories, Cat# A300-705A) was used for WB, and suitable for mice, which had been validated by Bethyl Laboratories.
Anti-HDAC2 (Cell Signaling Technology, Cat# 2545) was used for IP, and suitable for mice, which had been validated by company.
Anti-SATB2 (Abcam, Cat# ab92446) was used for WB, CHIP, IF and IP, and suitable for mice, which had been validated by both at Abcam and at Gu, W. et al, 2021.
Anti-SATB2 (Santa Cruz Biotechnology, Cat# sc-518006) was used for WB, and suitable for mice, which had been validated by company.
Anti-CA1 (Abcam, Cat# Ab108367) was used for IF, and suitable for mice, which had been validated at Gu, W. et al, 2021.
Anti-FLAG Agarose Gel (Sigma-Aldrich, Cat# A2220) was used for IP, and suitable for detecting overexpressed FLAG tag protein, which had been validated by company.
Anti-V5 Tag (Invitrogen, Cat# R960-25) was used for WB, and suitable for detecting overexpressed V5 tag protein in cells, which had been validated by company.
Anti-HA Tag (Cell Signaling Technology, Cat# 3724) was used for WB, and suitable for detecting overexpressed HA tag protein in cells, which had been validated by company.
Anti-MTTP (Sigma-Aldrich, Cat# HPA054862) used for IHC, was validated in human by company. The application of IHC in mice was validated in our studies.
Anti-HNF4A (Abcam, Cat# ab41898) was used for CHIP, and suitable for mice, which had been validated by company.
Anti-E-cadherin (Thermo Fisher Scientific, Cat# 13-1900) was used for IF, and suitable for mice, which had been validated by company.
Anti-H3K27Ac (Active Moti, Cat# 39135) was used for CHIP, and suitable for mice, which had been validated by company.
Anti-Rabbit IgG-HRP (Cell Signaling Technology, Cat# 7074) was used as a secondary antibody for WB which had been validated by company.
Anti-Mouse IgG-HRP (Cell Signaling Technology, Cat# 7076) was used as a secondary antibody for WB which had been validated by company.
Anti-Rabbit IgG ImmPRESS-HRP Polymer (Vector Laboratories, Cat# MP-7451) was used as a secondary amplifier for IHC which had been validated by company.

Eukaryotic cell lines

Policy information about cell lines and Sex and Gender in Research

Cell line source(s)	HEK293FT cell line was purchased from Thermo Fisher Scientific (#R70007).
Authentication	None of the cell line used have been authenticated.
Mycoplasma contamination	Tested negative
Commonly misidentified lines (See ICLAC register)	No commonly misidentified cell line was used in this study

Animals and other research organisms

Policy information about studies involving animals; ARRIVE guidelines recommended for reporting animal research, and Sex and Gender in Research

Laboratory animals	Mta2loxp/loxp (Xiangdong Lu et al. 2008) A gift from Dr. Robert G. Roeder of the Rockefeller University and Dr. Yi Zhang of Harvard Medical School who originally made the strain); Vil-CreERT2 strain ((el Marjou et al., 2004) A gift from Sylvie Robine of Institute Pasteur). To confer conditional deletion of MTA2, 4 mg per 25 g of body weight of tamoxifen (TAM, 10 mg per ml in corn oil) was intraperitoneally injected into both Mta2cKO (Vil-CreERT2; Mta2f/f) and Ctrl (Mta2f/f) mice (male and female equally, at 2 months old) once every 2 days for a total of 3 times. Mice were housed and bred in an ambient temperature (18°C -22°C) - and humidity (50%-60%)- controlled environment with 12 hours light/dark cycle and food/water supplement.
Wild animals	No wild animals used in this study
Reporting on sex	All animal experiments were performed with male and female mice equally.
Field-collected samples	No field-collected samples used in this study
Ethics oversight	All experiments were were conducted under the IACUC protocol 2018-0050 at Weill Cornell Medical College.

Note that full information on the approval of the study protocol must also be provided in the manuscript.

Plants

Seed stocks	n/a
Novel plant genotypes	n/a
Authentication	n/a

ChIP-seq

Data deposition

☒ Confirm that both raw and final processed data have been deposited in a public database such as GEO.

☒ Confirm that you have deposited or provided access to graph files (e.g. BED files) for the called peaks.

Data access links<br>*May remain private before publication.*	https://www.ncbi.nlm.nih.gov/geo/query/acc.cgi?acc=GSE213877<br>https://www.ncbi.nlm.nih.gov/geo/query/acc.cgi?acc=GSE245751
Files in database submission	ChIPseq raw data and processed BigWig data.
Genome browser session<br>(e.g. UCSC)	*Provide a link to an anonymized genome browser session for "Initial submission" and "Revised version" documents only, to enable peer review. Write "no longer applicable" for "Final submission" documents.*

Methodology

Replicates	2-3
Sequencing depth	≥ 20 million read pairs per sample
Antibodies	Rabbit monoclonal anti SATB2 (WB, ChIP, IF and IP) Abcam Cat# ab92446, RRID: AB_10563678<br>Rabbit polyclonal anti MTA2 Abcam Cat# ab8106, RRID: AB_306276<br>Mouse monoclonal anti HNF4A Abcam Cat# ab41898, RRID: AB_732976<br>Rabbit polyclonal anti H3K27Ac, Active Motif, Cat# 39135, RRID: AB_2614979
Peak calling parameters	Reads were mapped using Bowtie2 version 2.4.1 to mm10 genome; Peaks were called using callpeak function in the MACS2 package with the following parameters (callpeak -t ChIPfile.bam -c Inputfile.bam --format Paired-end BAM --gsize M.musculs(1.87e9) -m 10 30 -q 0.001-bw 300); bigwigs made using bamCoverage function in the deeptools v3.4.3 with following the parameters (bamCoverage --binSize 25 -p 4 --normalizeUsing RPKM --extendReads $fragLength -b Chip.bam -o ChIP.bw)
Data quality	We used peaks showing q-val < 0.001 and mfold enrichment 10 to 30 and used ChIP-seq from Inputs or knockouts as controls.
Software	Bowtie2 version 2.4.1 for read mapping, MACS2 (for calling peaks), .deeptools v3.4.3 for generating bigwigs and Integrated Genomics Viewer v2.16.2 for visualizing bigwigs.
